# Non-viral-mediated suppression of AMIGO3 promotes disinhibited NT3-mediated regeneration of spinal cord dorsal column axons

**DOI:** 10.1038/s41598-018-29124-z

**Published:** 2018-07-16

**Authors:** Sharif Almutiri, Martin Berry, Ann Logan, Zubair Ahmed

**Affiliations:** Neuroscience and Ophthalmology, Institute of Inflammation and Ageing, University of Birmingham, Edgbaston, Birmingham, B15 2TT UK

## Abstract

After injury to the mature central nervous system (CNS), myelin-derived inhibitory ligands bind to the Nogo-66 tripartite receptor complex expressed on axonal growth cones, comprised of LINGO-1 and p75^NTR^/TROY and induce growth cone collapse through the RhoA pathway. We have also shown that amphoterin-induced gene and open reading frame-3 (AMIGO3) substitutes for LINGO-1 and can signal axon growth cone collapse. Here, we investigated the regeneration of dorsal root ganglion neuron (DRGN) axons/neurites after treatment with a short hairpin RNA (sh) AMIGO3 plasmid delivered with a non-viral *in vivo*-jetPEI vector, and the pro-survival/axogenic neurotrophin (NT) 3 *in vitro* and *in vivo*. A bi*cis*tronic plasmid, containing both shAMIGO3 and NT3 knocked down >75% of AMIGO3 mRNA in cultured DRGN and significantly overexpressed NT3 production. *In vivo*, intra-DRG injection of *in vivo*-jetPEI plasmids containing shAMIGO3/*gfp* and shAMIGO3/*nt3* both knocked down AMIGO3 expression in DRGN and, in combination with NT3 overexpression, promoted DC axon regeneration, recovery of conduction of compound action potentials across the lesion site and improvements in sensory and locomotor function. These findings demonstrate that *in vivo*-jetPEI is a potential non-viral, translatable DRGN delivery vehicle *in vivo* and that suppression of AMIGO3 disinhibits the growth of axotomised DRGN enabling NT3 to stimulate the regeneration of their DC axons and enhances functional recovery.

## Introduction

Adult mammalian central nervous system (CNS) axons do not regenerate after injury probably because ontogenetic axogenic intracellular signalling is suppressed during CNS maturation. Axon growth inhibitory ligands also become incorporated into the maturing CNS neuropil, derived from both myelin and the incipient scar, which further limits CNS axon regeneration^1^. The former comprises Nogo-A, myelin associated glycoprotein (MAG), and oligodendrocyte-derived myelin glycoprotein (OMgp), while the latter comprise chondroitin sulphate proteoglycan (CSPG), NG2, semaphorins and ephrins (secreted by reactive astrocytes and invading meningeal fibroblasts)^1–6^. After binding to their cognate receptors, myelin- and scar-derived inhibitory ligands activate intracellular signals which converge on the RhoGTPase pathway, mediating axon growth cone collapse^1–6^. Myelin-derived inhibitors bind to a tripartite receptor complex comprised of NGR1/p75^NTR^/LINGO-1 in which TROY^4,7^ substitutes for p75^NTR^ and AMIGO3 (amphoterin-induced gene and open reading frame-3) for LINGO1 in the immediate post-injury period^8^. Evidence for the latter proposition is derived from experiments in which: *in vitro* (i), knockdown of AMIGO3 permits retinal ganglion cell (RGC) and dorsal root ganglion neuron (DRGN) neurites to grow on a CNS myelin extract (CME) substrate; (ii), RhoA is activated in response to co-transfection with *amigo3/ngr1/p75*^*ntr*^; and (iii), significant numbers of neurites grow after knockdown of AMIGO3; and (iv), *in vivo*, AMIGO3, not LINGO-1, becomes elevated in RGC/DRGN for up to 10 days after optic nerve/dorsal column (DC) crush injury^8^.

Viral vectors are commonly used to transfect CNS cells with shRNA to inhibit target mRNA translation^9–11^. Viral vectors, such as adeno-associated virus (AAV), transfect multiple genes but are restricted by the insert capacity whilst shRNA delivery delays suppression of targeted mRNA by 14–28 days. Both these disadvantages retard the therapeutic effect and reduce their translational potential^12–14^. Another significant impediment to the use viral vectors *in vivo* is that maximum transgene expression requires 7–14 days and hence viral vector transfection is limited in acute conditions. Non-viral gene delivery vectors include cationic lipid agents and a more recently formulated non-lipid polymer, polyethylenimine (*in vivo*-jetPEI), which transfects cells both *in vitro* and *in vivo*^15,16^. *In vivo*-jetPEI has high transfection efficiency and yields up to 4× higher transfection rates than naked DNA^15^. Moreover, *in vivo*-jetPEI is easy to prepare, stable and safe, and *in vivo*-jetPEI/shRNA transfection induces faster suppression of mRNA translation than viral vectors, giving immediate therapeutic benefit^17^. For example, in the CNS, Melanopsin expression is rapidly abolished in RGC after intravitreal injection of *in vivo*-jetPEI-shMelanopsin^18^.

In this study, we show that *in vivo*-jetPEI successfully delivers a bi*cis*tronic plasmid DNA to DRGN encoding shAMIGO3 to suppress AMIGO3 and perturb axon growth inhibitory signalling while, at the same time inducing the overexpression of neurotrophin-3 (NT3). We show that suppression of AMIGO3 and concomitant stimulation of DRGN with NT3 enhanced disinhibited DRGN neurite/axon regeneration and improved functional recovery. These results suggest that *in vivo*-jetPEI PEI delivers genes to DRGN without the disadvantages of viral vectors, rendering *in vivo*-jetPEI PEI as translationally relevant.

## Results

### *In vitro* experiments

#### *In vivo*-jetPEI delivered plasmids knock down AMIGO3 mRNA

**Figure 1:**
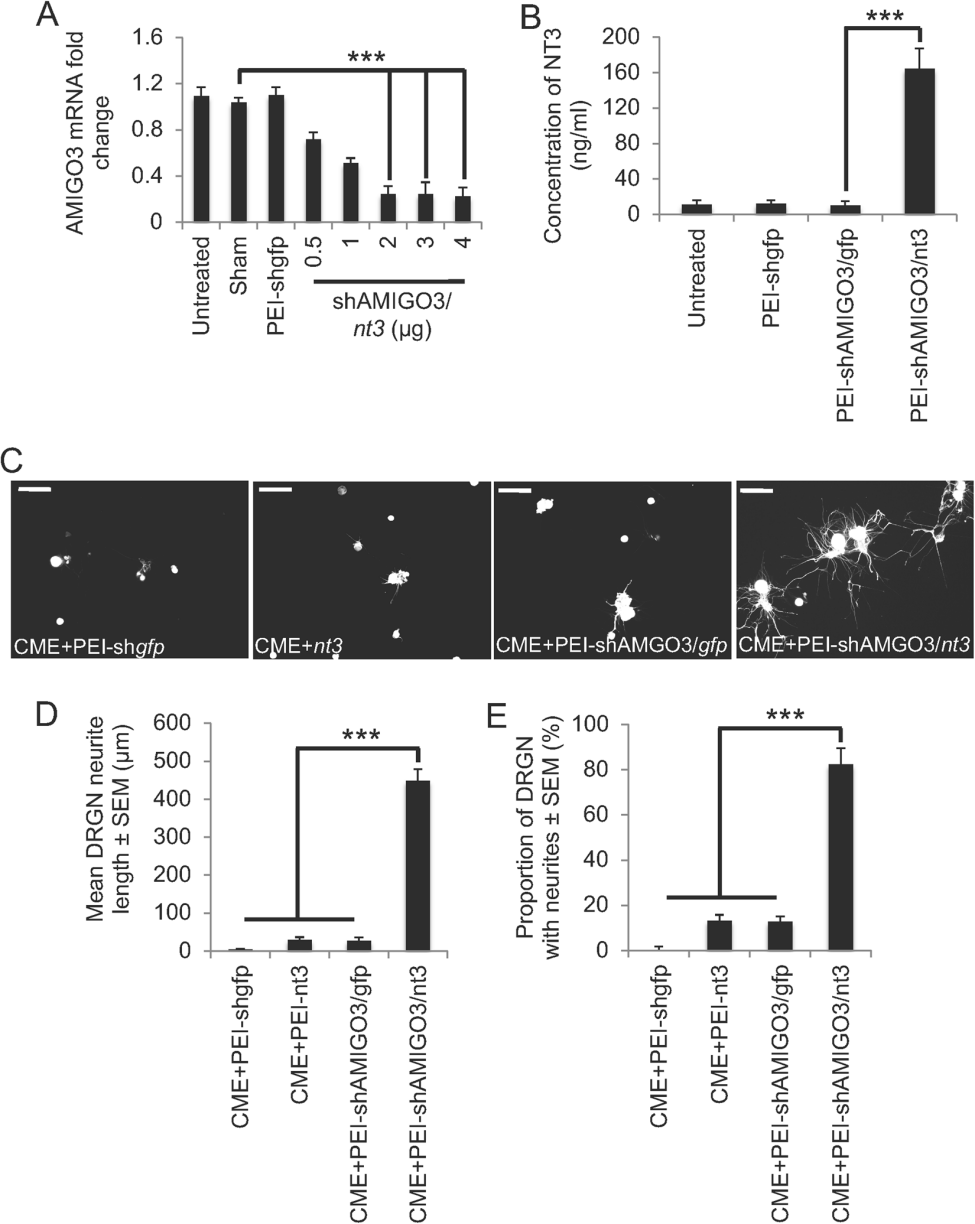
Knockdown of AMIGO3 and NT3 over-expression by PEI-delivered plasmid DNA disinhibited DRGN neurite outgrowth. (**A**) Increasing concentrations of plasmid DNA encoding shAMIGO3/*nt3* efficiently suppressed AMIGO3 mRNA in cultured DRGN. (**B**) Plasmids encoding *nt3* significantly increased the titres of NT3 in DRGN culture media. (**C**) Representative images show that in the presence of CME, plasmid DNA encoding *gfp*, *nt3* or shAMIGO3/*gfp* did not, but that plasmids encoding shAMIGO3 and *nt3* did promote DRGN neurite outgrowth. *Note:* DRGN do not have neurites due to the presence of inhibitory concentrations of CME, which does not affect their survival. (**D**) Quantification of the mean DRGN neurite length and (**E**) the proportion of DRGN with neurites showed that AMIGO3 suppression combined with *nt3* overexpression promoted significant disinhibited DRGN neurite outgrowth. Scale bars in C = 50 μm. ***P < 0.0001, ANOVA.

In untreated, sham and non-specific sh*gfp* control transfected DRGN, there was no change in mRNA for AMIGO3 suggesting that none of these treatments had any non-specific effects on *amigo3* mRNA (Fig. 1A). Treatment with increasing amounts of shAMIGO3 plasmid delivered by *in vivo*-jetPEI (referred to as PEI in the results section from herein) caused a dose-dependent decrease in *amigo3* mRNA to a minimum at 2 µg of plasmid DNA, correlating with 80% knockdown compared to untreated, sham or sh*gpf* controls (Fig. 1A). Increasing the amount of plasmid DNA above 2 µg did not decrease *amigo3* mRNA levels further, confirming that 2 µg of plasmid DNA gave optimal knockdown.

#### PEI delivered shAMIGO3/*nt3* plasmids increased NT3 secretion into the culture media

In untreated, sham, non-specific PEI-sh*gfp* and PEI-shAMIGO3/*gfp*-treated DRGN, little or no NT3 was detected by ELISA in culture supernatant (Fig. 1B). However, in culture media from DRGN, treated with 2 µg of PEI-shAMIGO3/*nt3* plasmid DNA significant production and release of NT3 occurred (164 ± 24 ng/ml, P < 0.0001) compared to PEI-shAMIGO3-*gfp* (Fig. 1B). These results suggest that 2 µg of plasmid DNA was optimal for *amigo3* mRNA knockdown and NT3 production.

#### Knockdown of AMIGO3 and concomitant stimulation of NT3 disinhibited DRGN neurite outgrowth

In PEI-sh*gfp*-, PEI-*nt3* or PEI-shAMIGO3/*gfp*-treated cultures, little or no DRGN neurite outgrowth was observed on CME substrates (Fig. 1C–E). However, treatment of cultures with PEI-shAMIGO3/*nt3* significantly increased both neurite length (448 ± 31 µm, P < 0.0001 compared to PEI-shAMIGO3/*gfp*), and the proportion of DRGN with neurites (82 ± 8%, P < 0.0001 compared to PEI-shAMIGO3/*gfp*; Fig. 1C–E). Thus, delivery of shAMIGO3/*nt3* plasmids by PEI significantly enhanced DRGN neurite outgrowth on a CME substrate.

### *In vivo* experiments

#### PEI-shAMIGO3 enhanced transduction in all sizes of DRGN

**Figure 2:**
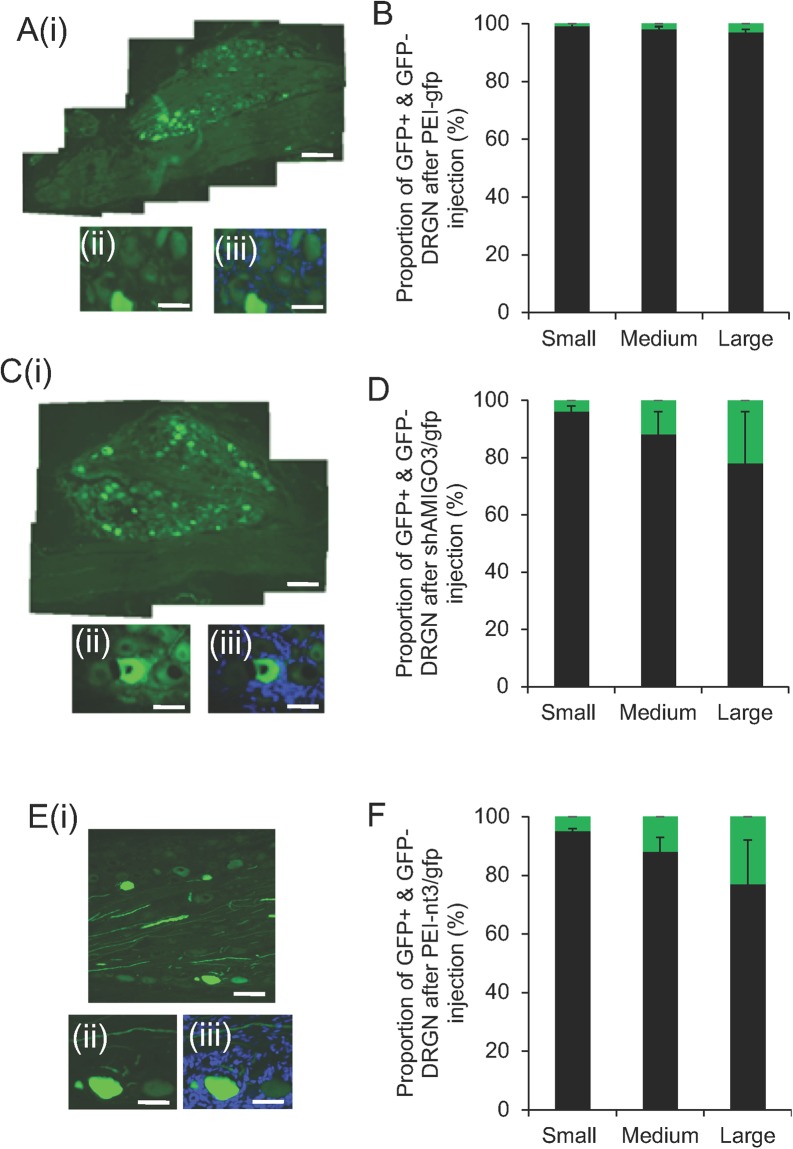
GFP^+^ (green) DRGN in DRG in the DC + PEI-*gfp* group (**A**(**i**,**ii**)); high power to show GFP^+^ DRGN, (**iii**); high power GFP^+^ DRGN with DAPI counterstained (blue) nuclei), (**B**) the proportion of GFP^+^ small (0–29 μm), medium (30–59 μm) and large (>60 μm) diameter DRGN (green bar); % total GFP^+^ + GFP^−^ small, medium and large DRGN (black bar). (**C**) GFP^+^ DRGN in the DC + PEI-shAMIGO3*/gfp* group (**C**(**i**),(**ii**)), high power to show GFP^+^ DRGN, (**iii**); high power GFP^+^ DRGN with DAPI counterstained (blue) nuclei), (**D**) the proportion of GFP^+^ small (0–29 μm), medium (30–59 μm) and large (>60 μm) diameter DRGN (green bar); % total GFP^+^/GFP^−^ small, medium and large diameter DRGN (black bar). (**E**) GFP^+^ DRGN in the DC + PEI-nt3*/gfp* group (**E**(**i**),(**ii**)), high power to show GFP^+^ DRGN, (**iii**); high power GFP^+^ DRGN with DAPI counterstained (blue) nuclei), (**F**) the proportion of GFP^+^ small (0–29 μm), medium (30–59 μm) and large (>60 μm) diameter DRGN (green bar); % total GFP^+^/GFP^−^ small, medium and large diameter DRGN (black bar). Scale bar in (**A**(**i**)), (**C**(**i**)) and (**E**(**i**)) = 400 μm, in (**A**(**ii**)), (A(**iii**)), (**C**(**ii**)) and (**C**(**iii**)), (**E**(**ii**)) and E(iii) = 100 μm.

No GFP^+^ DRGN was observed in either intact controls (IC) or in dorsal column (DC) crush injured animals (not shown). In the DC + PEI-*gfp* (Fig. 2A(i–iii),B), DC + PEI-*nt3*/*gfp* (not shown) and DC + PEI-shAMIGO3/*nt3* groups, similar numbers of DRGN were GFP^+^ (green) (Fig. 2C(i–iii),D). High power insets of GFP (Fig. 2A(ii),C(ii)) and images merged with DAPI counterstain (blue) (Fig. 2A(iii),C(iii)) showed variable weak and high levels of GFP^+^ DRGN. Approximately 1, 2 and 3% of small, medium and large diameter DRGN were GFP^+^, respectively, in the DC + PEI-*gfp* group (Fig. 2B) and, in the DC + PEI-shAMIGO3/*gfp* group, GFP expression increased significantly to 4, 12 and 22% in small, medium and large diameter DRGN, respectively (Fig. 2D). Similar levels of DRGN transduction were also observed in DC + PEI-nt3/*gfp* groups (Fig. 2E,F). These results suggested that PEI delivered plasmids encoding shAMIGO3-*/gfp* or nt3/*gfp* enhanced transduction in all sizes of DRGN compared to PEI-*gfp* alone.

#### PEI/shAMIGO3 down regulated AMIGO3 in DRGN *in vivo*

**Figure 3:**
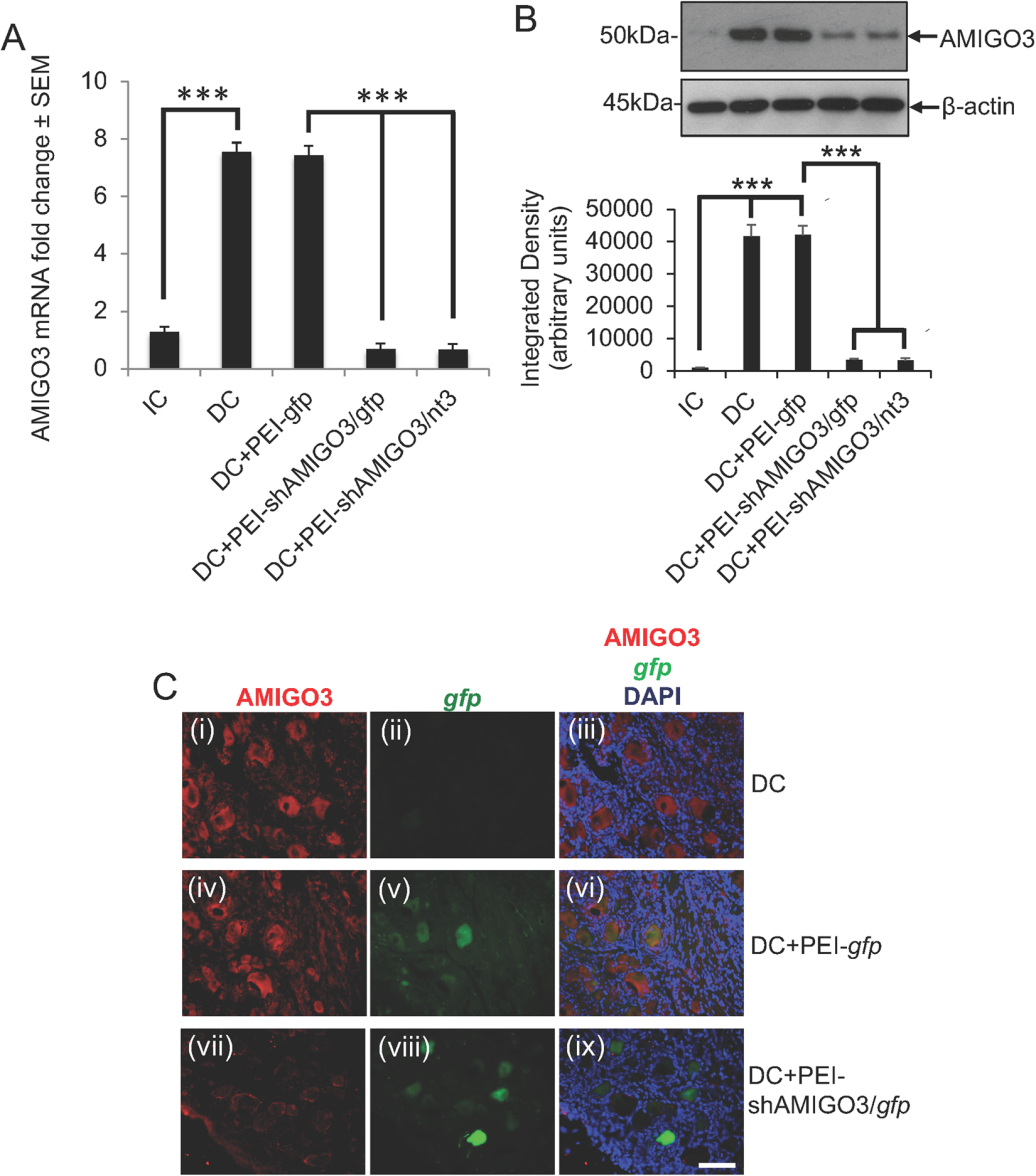
AMIGO3 levels are suppressed in DRGN after injection of PEI transduced plasmids encoding shAMIGO3. (**A**) Low levels of AMIGO3 mRNA in IC increased significantly after DC injury (P < 0.0001) and remained high in the DC + PEI-*gfp* group. AMIGO3 mRNA levels reduced significantly in both DC + PEI/shAMIGO3/*gfp* and DC + PEI-shAMIGO3/*nt3* groups (P < 0.0001). (**B**) Western blot and subsequent densitometry for AMIGO3 protein reflected the changes in mRNA. β-actin was used as a loading control (full scans shown). (**C**) Immunohistochemistry for AMIGO3 showed high levels of AMIGO3 (red) in DRGN after DC (**C**(**i**)), which were *gfp*^−^ (**C**(**ii**)). AMIGO3 levels remained high in DC + PEI-*gfp* groups (**C**(**iv**)), with *gfp* expression (green) in some DRGN (**C**(**v**)). AMIGO3 levels were suppressed in DRGN from DC + PEI-shAMIGO3/*gfp* groups (**C**(**vii**)), with *gfp* expression in some DRGN (**C**(**viii**)). (**C**(**iii**)), (**C**(**vi**)) and (C(**ix**)) are merged images showing AMIGO3 (red), *gfp* (green) and DAPI stained nuclei (blue). Scale bar = 100 μm. ***P < 0.0001, ANOVA.

The levels of *amigo3* mRNA increased significantly by 7.54 ± 0.33 and 7.42 ± 0.34-fold (P < 0.0001) in both DC and DC + PEI-*gfp* groups compared to those observed in the IC group (Fig. 3A). However, *amigo3* mRNA levels were suppressed by nearly 11-fold in DRG treated with both PEI-shAMIGO3/*gfp* and PEI-shAMIGO3/*nt3* (P < 0.0001 compared to DC + PEI-*gfp*; Fig. 3A). Western blot and subsequent densitometry reflected these significant increased changes (P < 0.0001) in AMIGO3 protein in DC and DC + PEI-gfp groups whilst a significant reduction (P < 0.0001) was observed in DC + PEI-shAMIGO3/*gfp* and DC + PEI-shAMIGO3/*nt3* groups (Fig. 3B). Immunohistochemistry also detected high levels of AMIGO3 (red) in DC and DC + PEI-*gfp*-treated DRG in GFP^+^ (green) and GFP^−^ DRGN (Figs 3C(i)-(iii) and 4C(iv)-(vi), respectively; panels (iii) and (vi) show merged images with DAPI^+^ nuclei stained in blue). In the DC-PEI-*gfp* group (Fig. 3C(iv)-(vi)) almost 30% of the DRGN were GFP^+^/AMIGO3^+^. In the DC + PEI-shAMIGO3/*gfp* group, similar numbers of DRGN as in the DC + PEI-*gfp* group were GFP^+^ but few DRGN were AMIGO3^+^ (Fig. 3C(vii)-(ix); panel (ix) = merged with DAPI^+^ nuclei stained in blue). These results demonstrate that: (1), PEI-delivered plasmids encoding shAMIGO3 efficiently knocked down AMIGO3 protein in DRGN; and (2), knockdown of AMIGO3 occurred in both transfected and un-transfected DRGN.

#### NT3 levels are overexpressed in DRGN after PEI-shAMIGO3/nt3 transfection

**Figure 4:**
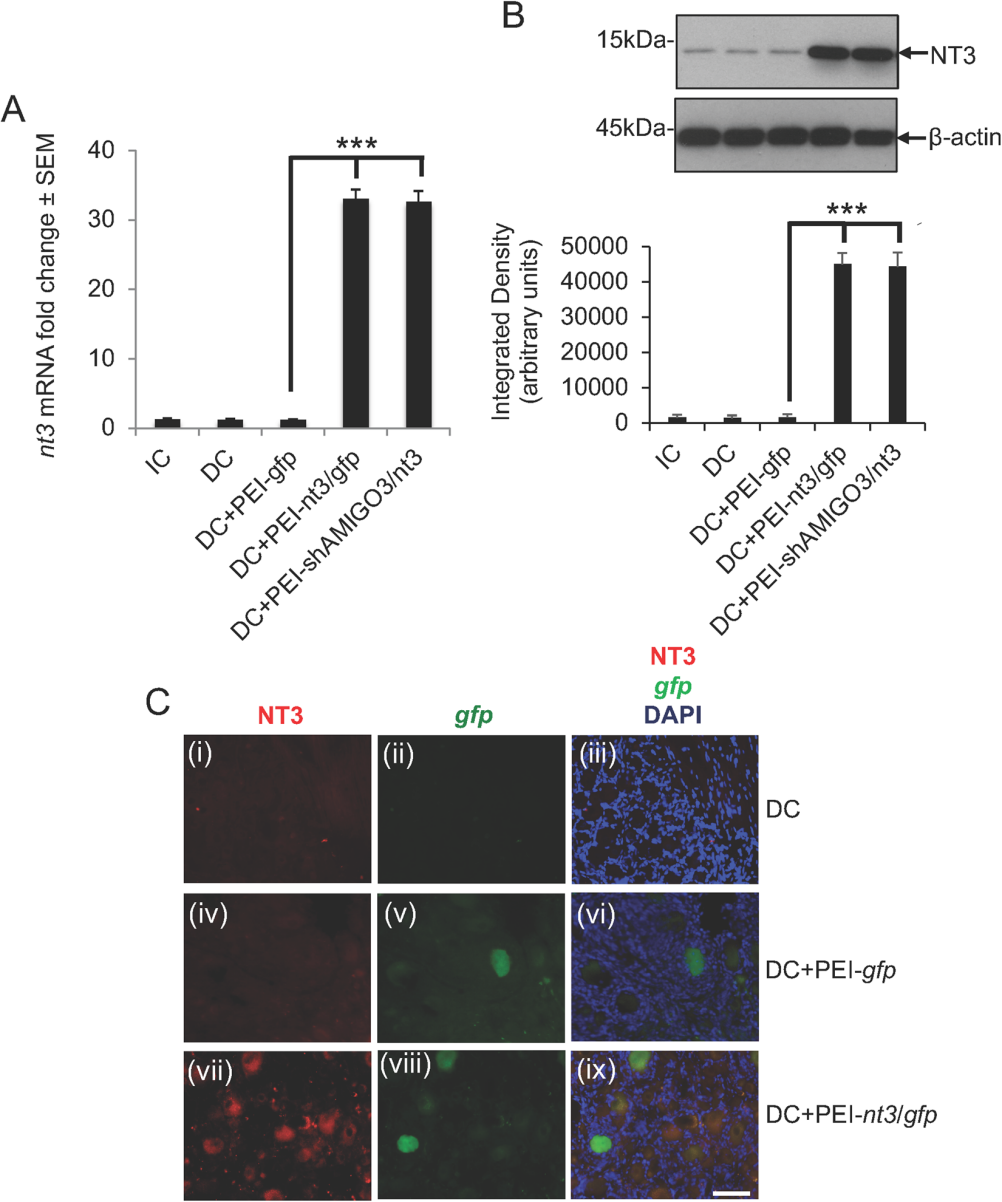
NT3 levels were overexpressed in DRGN after injection of *in vivo-jet*PEI transduced plasmids encoding shAMIGO3. (**A**) Low levels of *nt3* mRNA were detected in IC, DC and DC + PEI-*gfp* groups, whilst significantly higher levels (P < 0.0001) were detected in DC + PEI-nt3/*gfp* and DC + PEI-shAMIGO3/*nt3* groups. (**B**) Western blot and subsequent densitometry for NT3 protein reflected the changes in mRNA. β-actin was used as a loading control (full scans shown). (**C**) Immunohistochemistry for NT3 showed low levels of NT3 (red) in DRGN after DC. (**C(i)**), which were *gfp*^−^ (**C(ii)**) and in DC + PEI-*gfp* groups (**C**(**iv**)), with *gfp* expression (green) in some DRGN (**C(v)**). NT3 levels (**Cvii**)) were high in both gfp^+^ and gfp^−^ DRGN (**C**(**viii**)). Scale bar = 100 μm. C(iii), C(vi) and C(ix) are merged images showing NT3 (red), *gfp* (green) and DAPI stained nuclei (blue). ***P < 0.0001, ANOVA.

The levels of *nt3* mRNA remained low or unchanged in IC, DC and DC + PEI-*gfp*-treated DRGN (Fig. 4A). However, in DRGN treated with DC + PEI-*nt3*/*gfp* and DC + PEI-shAMIGO3/*nt3*, *nt3* mRNA levels increased significantly and by 33.0 ± 1.35-fold, compared to DC + PEI-*gfp*-treated DRGN (P < 0.0001; Fig. 4A). Similar changes in the levels of NT3 protein were corroborated by western blot and subsequent densitometry showing high levels of NT3 production in DC + PEI-*nt3*/*gfp* and DC + PEI-shAMIGO3/*nt3*-treated groups (Fig. 4B). Little or no NT3 immunoreactivity (red) was present in GFP^+^ (green) and GFP^−^ DRGN in IC (not shown), DC (Fig. 4C(i)-(iii)) and DC + PEI-*gfp* (Fig. 4C(iv)-(vi); panels (iii) and (vi) show merged images with DAPI^+^ nuclei stained in blue) groups. However, in the DC + PEI-*nt3*/*gfp* group (Fig. 4C(vii)-(ix); panel (ix) = merged with DAPI^+^ nuclei stained in blue), high levels of NT3 immunoreactivity were observed in both GFP^+^ and GFP^*−*^ DRGN. These results demonstrate that: (1), PEI successfully transfected DRGN with plasmids encoding *nt-3* resulting in NT3 protein expression; and (2), NT3 was detected in both transfected and un-transfected DRGN.

#### PEI-shAMIGO3/nt3 transfected axotomised DRGN regenerate axons in the DC

**Figure 5:**
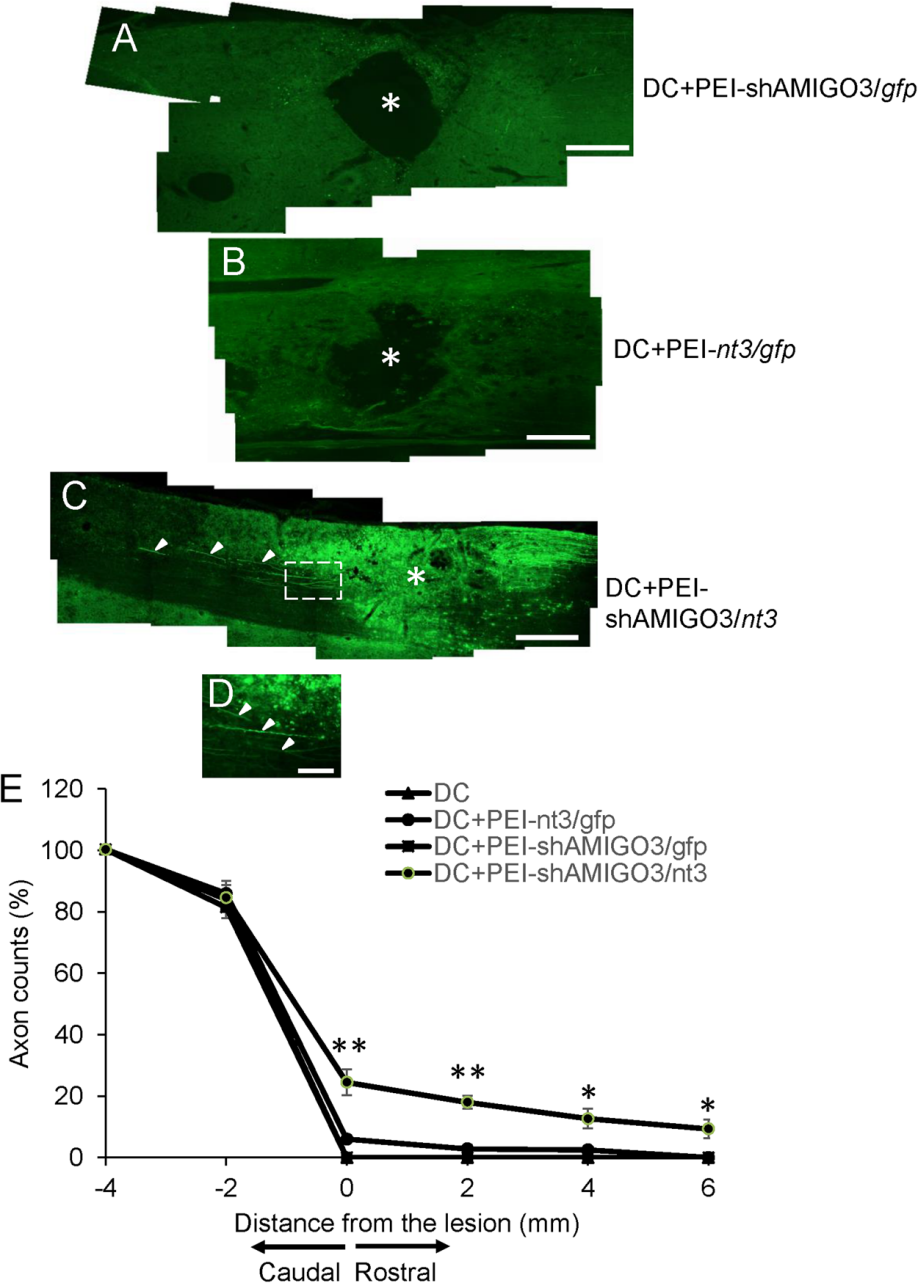
Suppression of AMIGO3 in DRGN promoted axon regeneration in the cord rostral to the lesion. There were no GAP43^+^ regenerating axons in the DC of the (**A**) PEI + shAMIGO3/*gfp* and (**B**) DC + PEI-*nt3*/*gfp* groups with a large cavity (*) present at the lesion site. (**C**) Knockdown of AMIGO3 and co-incident up-regulation of NT3 in the DC + shAMIGO3/*nt3* group promoted GAP43^+^ DRGN axon (arrowheads) regeneration through the DC lesion site. ((**D**), high power view of axons coursing rostrally from the lesion site (arrowheads)), with the absence of a cavity in the lesion site. Scale bar in A–C = 500 μm; in D = 50 μm. (**E**) GAP43^+^ axon fiber counts at specific distances rostral and caudal to the lesion site showed a significant proportion of axons present at 2, 4 and 6 mm rostral to the lesion site. **P < 0.001, ANOVA; * < 0.05, ANOVA.

DC injury induced a large cavity at the lesion site (*) in the cords of DC and DC + PEI/*gfp* (not shown), DC + PEI-shAMIGO3/*gfp* (Fig. 5A) and DC + PEI-*nt3/gfp* (Fig. 5B) groups; few immunoreactive GAP43^+^ axons were seen (Fig. 5A,B). By contrast, in the injured cords of the DC + PEI-shAMIGO3/*nt3* groups, no cavities developed and many regenerating GAP43^+^ axons crossed through caudal cord segment and some traversed the lesion site (*), growing for long distances into the rostral segment of the spinal cord (Fig. 5C (arrowheads), Fig. 5D; high power magnification of boxed region in Fig. 5C). Quantification of the number of GAP43^+^ DC axons regenerating through the lesion site shows that 24.5 ± 4.2, 18.0 ± 2.1, 12.7 ± 3.3 and 9.3 ± 3.1% of axons regenerated rostrally at 0, 2, 4 and 6 mm beyond the lesion site, respectively, in DC + PEI-shAMIGO3/*nt3* whilst no axons were present rostrally in either DC, DC + PEI-*nt3/gfp* or DC + PEI-shAMIGO3/*gfp*. These results demonstrate that PEI-shAMIGO3/*nt3* plasmids promote DC axon regeneration after DC injury.

#### PEI-delivered plasmids did not invoke off-target effects after intra-DRG injection

**Figure 6:**
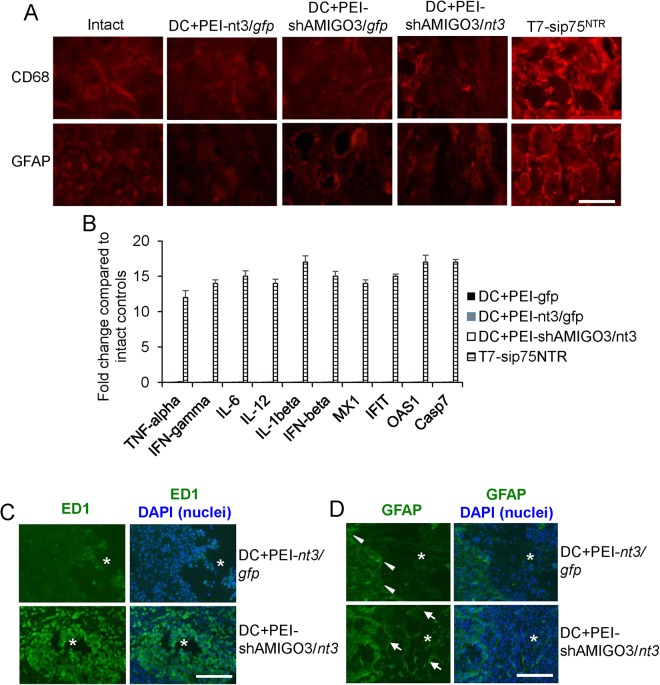
Response to PEI-delivered plasmids. (**A**) CD68 and GFAP immunoreactivity in intact and after PEI-delivered plasmids in section of DRG to show a lack of induction of immunological responses to these markers. In contrast, T7-sip75^NTR^ invoked significant changes in CD68 and GFAP^+^ immunoreactivity in DRG. (**B**) Lack of activation of off-target cytokines and innate immunity related genes in DRG after PEI-delivered plasmid transduction in DRG. Once again, T7-sip75^NTR^ showed significant upregulation of mRNA for all of these genes. (**C**) ED1 immunoreactivity was low in DC + PEI-nt3/*gfp*-treated animals whilst abundant ED1^+^ cells were present within the lesion site (*) in DC + PEI-shAMIGO3/*nt3*-treated animals. (**D**) GFAP^+^ astrocytes were restricted to the lesion edge in DC + PEI-nt3/*gfp*-treated animals whilst GFAP^+^ astrocytes invaded the lesion site in DC + PEI-shAMIGO3/*nt3*-treated animals. Scale bars in A = 100 µm; C and D = 500 µm.

Intra-DRG injection of any of the plasmids that were delivered by PEI did not invoke changes in the CD68 and GFAP immunoreactivity in DRG (Fig. 6A). In contrast, intra-DRG injection of T7-sip75^NTR^, a chemically synthesized siRNA that induces off-target effects^19^, invoked a marked increase in CD68 and GFAP immunoreactivity in DRG (Fig. 6A). Likewise, intra-DRG injection of PEI delivered plasmids did not invoke off-target immune-mediated cytokines or innate immunity genes such as TNF-α, IFN-γ, IL-6, IL-12/IL-23, IL-1β, and IFN-β, MX1, IFIT, OAS1 or Casp7 (Fig. 6B). In contrast, intra-DRG injection of sip75^NTR^ induced 12–17-fold increases in the mRNA of all of these genes (Fig. 6B). These results suggest that PEI-delivered plasmids do not invoke off-target effects in DRGN.

#### High numbers of ED1^+^ cells and astrocyte invasion into the lesion site were observed in DC + PEI-shAMIGO3/*nt3* treatment

In DC (not shown) and DC + PEI-*nt3/gfp-*treated groups, ED1^+^ immunoreactivity (green) was largely confined to the lesion (*demarcates lesion site) cavity edge (Fig. 6C) whilst in DC + PEI-shAMIGO3/*nt3*-treated groups, large numbers of ED1^+^ cells (green) were localized within the lesion core along with other non-ED1^+^ cells (DAPI stained nuclei; blue) (Fig. 6C). GFAP^+^ astrocytes (green) were also restricted to the edges of the lesion cavity in DC (not shown) and DC + PEI-*nt3/gfp-*treated groups (Fig. 6C; arrowheads show GFAP^+^ astrocytes at the lesion cavity edge). In contrast, GFAP^+^ (green) astrocytes (arrows) invaded the lesion core in DC + PEI-shAMIGO3/*nt3*-treated groups and were present amongst other cells (DAPI stained nuclei; blue) (Fig. 6D).

#### PEI-shAMIGO3/*nt3* transfection of axotomised DRGN improved compound action potentials and sensory and locomotor function

**Figure 7:**
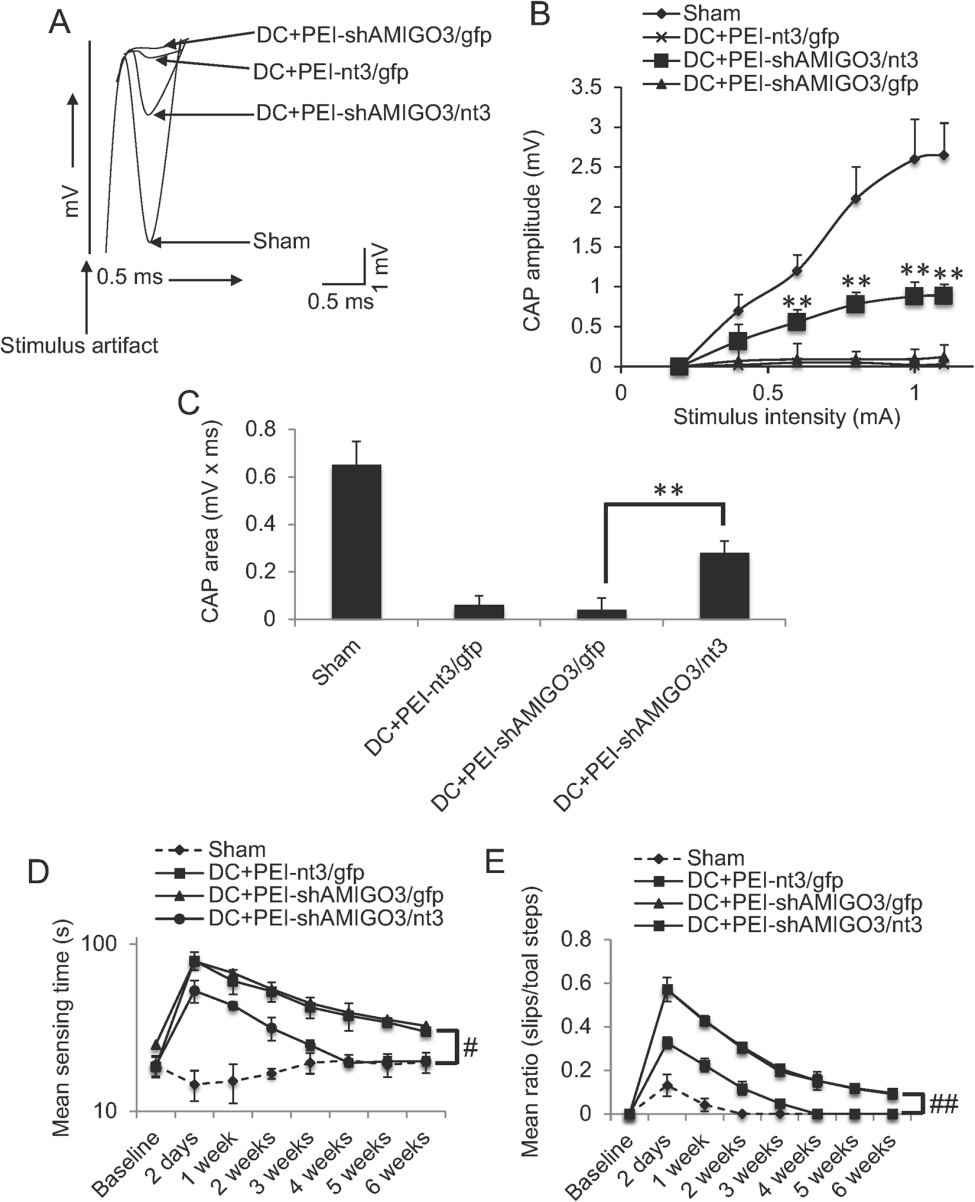
Suppression of AMIGO3 in DRGN preserved the spinal compound action potentials (CAP) across the lesion site. (**A**) Superimposed CAP traces from representative Sham controls, DC + PEI-shAMIGO3/*gfp* DC + PEI-*nt3*/*gfp* and DC + shAMIGO3/*nt3* groups. (**B**) Compared to Sham controls, negative CAP amplitudes (mV) were highly attenuated in DC + PEI-shAMIGO3/*gfp* and DC + PEI-*nt3*/*gfp* groups but was significantly improved in DC + PEI-shAMIGO3/*nt3* groups (P < 0.001, ANOVA). (**C**) Mean CAP area at different stimulus intensities recorded from Sham controls was significantly attenuated in DC + PEI-shAMIGO3/*gfp* but improved significantly in DC + PEI-shAMIGO3/*nt3* compared to DC + PEI-shAMIGO3/*gfp* and DC + PEI-*nt3*/*gfp* groups (**P < 0.001, ANOVA). (**D**) Mean sensing time for the tape removal test showed return to baseline Sham control levels by 4-wekks after DC and treatment with DC + PEI-shAMIGO3/*nt3* compared to other groups. (**E**) Mean error ratio to show the number of slips *versus* total number of steps in the horizontal ladder walking test. Once again DC + PEI-shAMIGO3/*nt3*-treated animals returned to Sham control levels by 4 weeks after DC injury. ^##^P < 0.012, generalised linear mixed models; ^#^P < 0.0011, linear mixed models.

Superimposed CAP traces from representative Sham control, DC + PEI-*nt3/gfp*, DC + PEI-shAMIGO3/*gfp* and DC + shAMIGO3/*nt3* groups show that in the DC + PEI-*nt/gfp3* and DC + PEI-shAMIGO3/*gfp* group, the negative CAP wave was significantly attenuated in amplitude compared to Sham controls, whereas in PEI-shAMIGO3/*nt3*, the negative amplitude increased markedly (Fig. 7A). The mean CAP amplitude was reduced after DC, compared to Sham controls (Fig. 7B). Significantly larger CAP amplitude was observed in DC + PEI-shAMIGO3/*nt3*-treated animals at all stimulation intensities, compared to DC + PEI-*nt3/gfp* and DC + PEI-shAMIGO3/*gfp* treatment groups (P < 0.001; Fig. 7B). In Sham controls, CAP area (0.51 ± 0.06 mV x ms) was reduced to 7.8 ± 9.8% of the CAP area in Sham controls (0.04 ± 0.05 mV x ms) (Fig. 7C). CAP area in DC + PEI-shAMIGO3/*nt3* was significantly larger (P < 0.001) than in DC + PEI-*nt/gfp3* and DC + PEI-shAMIGO3/*gfp* groups and was increased to 54.9 ± 9.8% of Sham control CAP area (Fig. 7C).

The mean sensing time for the tape removal test which assess sensory function^20^ remained between 12–25 s throughout the 6-week experimental time period in sham treated animals (Fig. 7D). The mean sensing time increased to 80–85 s in DC + PEI-*nt3* and DC + PEI-shAMIGO3/*gfp*-treated groups at 2d after DC lesion (Fig. 7D). However, in DC + PEI-shAMIGO3/nt3-treated animals, the mean sensing time at 2d was significantly lower (P < 0.001), taking 53 ± 8 s to detect the tape (Fig. 7D). The mean sensing time improved over the 6-week time period in all animals, however, in DC + PEI-shAMIGO3/*nt3*, the mean sensing time returned to sham treated baseline levels by 4 weeks after DC lesion. Over the 6-week time period, DC + PEI-shAMIGO3/*nt3*-treated animals were significantly better at detecting the tape and removing it compared to all other treatment groups (P < 0.0012, linear mixed model).

The mean ratio (slips/total steps) in the horizontal ladder crossing test, which detects locomotor function^20^ remained between 0.1 and 0 throughout the 6-week time period in sham treated animals (Fig. 7E). The mean ratio increased to 0.58 ± 0.05 in DC + PEI- in DC + PEI-*nt3* and DC + PEI-shAMIGO3/*gfp*-treated groups at 2d after DC lesion (Fig. 7E). However, in DC + PEI-shAMIGO3/nt3-treated animals, the mean ratio at 2d was significantly lower (P < 0.001), at 0.33 ± 0.02 (Fig. 7D). The mean ratio improved over the 6-week time period in all animals, however, in DC + PEI-shAMIGO3/*nt3*, the mean ratio returned to sham treated baseline levels by 4 weeks after DC lesion. Over the 6-week time period, DC + PEI-shAMIGO3/*nt3*-treated animals were significantly better at ladder crossing compared to all other treatment groups (P < 0.01, generalized linear mixed models).

Taken together, these results show that suppression of AMIGO3 and simultaneous NT3 stimulation of DC axon regeneration promotes sensory and locomotor function recovery.

## Discussion

In this study, we investigated the use of a non-viral vector for gene delivery to DRGN and showed *in vitro* and that shAMIGO3 knockdown combined with NT3 over-expression, induced DRGN to grow neurites on a CME substrate. We also showed *in vivo* that *in vivo*-jetPEI-delivered shAMIGO3/*gfp* plasmid DNA: (1), increased transduction in all sizes of DRGN; (2), efficiently knocked down AMIGO3 while simultaneously increasing NT3 production; (3), stimulated DC axon regeneration which conducted action potentials across the lesion site and led to enhanced sensory and locomotor function.

### *In vitro* findings

We tested the efficiency of *in vivo*-jetPEI-delivered plasmids to suppress AMIGO3 and upregulate NT3 in DRGN cultures. It has been recognised by us and others that a combinatorial strategy will be required to promote optimal CNS axon regeneration due to the complexity of the injury. For example, we have shown that suppression of axon growth inhibitory molecules combined with neurotrophic factor stimulation promotes significant disinhibited DRGN neurite outgrowth in the presence of CME^21–24^. In this study, 2 µg of plasmid DNA encoding shAMIGO3 was sufficient to significantly knock down AMIGO3, to similar levels that we observed with siAMIGO3^8^. The same amount of DNA was also able to promote over-expression of NT3 in DRGN and significantly increased titres of NT3 secretion into the culture medium.

However, similar to our previous observations with siAMIGO3, knockdown of AMIGO3 using an shAMIGO3 alone did not overcome CME-mediated neurite growth inhibition. Added simultaneous stimulation with NT3 to promote DRGN neurite outgrowth was required, confirming that disinhibition alone in insufficient to promote DRGN neurite outgrowth in the presence of CME but that concomitant stimulation of growth is required to drive regeneration^22,25,26^.

### *In vivo* findings

We compared the targeting efficiency of non-viral plasmids delivered to DRGN through intra-DRG injection with *in vivo*-jetPEI. There is a high heterogeneity of DRGN in size distribution^27,28^ and our plasmids, especially in the presence of shAMIGO3, increased transduction in all sizes of DRGN. In a previous study, we found that preferential targeting of large diameter DRGN was achieved with AAV8 delivered *gfp*^28^. It is possible that the large surface area exposed to the plasmids results in an increased incidence of transduction^28^. It is well established that the majority of the DRGN that project their axons through the DC are large diameter DRGN and hence their increased transduction rates by our plasmids is actually beneficial, after DC injury. It is clear from our study that NT3 production by DRGN through the local production of NT3 may also have paracrine effects on neighbouring DRGN, since both GFP^+^ (i.e. transduced DRGN) and GFP^−^ DRGN (i.e. non-transduced DRGN) were NT3^+^.

A common concern with RNA interference (RNAi) is RNAi-associated immune stimulation, which is a major hurdle to the safety and efficacy of RNAi. The induction of innate immunity may arise from sequence-dependent effects, delivery vehicles and the RNAi process itself leading to undesired effects and misinterpretation of the data^29,30^. However, our study found no evidence of changes in the most common off-target cytokines and innate immunity genes after transduction of DRGN with *in vivo*-jetPEI-delivered plasmids. Others have also reported that *in vivo*-jetPEI vector is safe and does not induce off-target immune-mediated cytokines such as TNF-α, IFN-γ, IL-6, IL-12/IL-23, IL-1β, and IFN-β^31^. Our study therefore suggests that *in vivo*-jetPEI may be safe for clinical applications.

Not only did the *in vivo*-jetPEI-delivered shAMIGO3 suppress AMIGO3 levels in >80% DRGN, immunoreactivity for AMIGO3 was reduced in both *gfp*^+^ and gfp^−^ DRGN. This suggests that the plasmid efficiently suppressed AMIGO3 levels in both transduced and non-transduced DRGN. At present, we cannot explain why non-transduced DRGN showed reduced AMIGO3 levels but, to our knowledge, this is the first report to show such an effect. It may suggest that suppression of AMIGO3 in DRGN had paracrine effects on neighbouring DRGN, representing an intriguing possibility that not all DRGN require targeting to promote therapeutically effective outcomes. Similarly, over-expression of NT3 was also apparent in both *gfp*^*+*^ and *gfp*^*−*^ DRGN suggesting that both transfected and non-transfected DRGN produced high titres of NT3. Our results therefore demonstrate that *in vivo*-jetPEI efficiently knocked down AMIGO3 and up-regulated NT3 in DRGN.

Knockdown of AMIGO3 and simultaneous stimulation with NT3 promoted significant DRGN axon regeneration after DC injury. This was correlated with significant improvements in CAP amplitudes across the lesion site and sensory and locomotor function. This is the first demonstration that knockdown of AMIGO3 promotes spinal cord axon regeneration *in vivo* and demonstrates that AMIGO3 may require targeting to promote CNS axon regeneration. We previously showed that AMIGO3 could substitute for LINGO-1 in binding to p75^NTR^ and NgR1 to induce RhoA activation in response to CME. Our current results are consistent with these findings. Axons regenerated for up to 6 mm rostral to the lesion site and conducted action potentials across the lesion site correlating with improved sensory and locomotor function. Other types of behavioural analyses however, have been shown to be inconclusive with the T8 DC lesion model, since measurable functional improvements in mechanical and thermal hyperplasia do not occur^32,33^.

Our current study demonstrates *in vivo* proof-of-principle that in DRGN cultures grown in the presence of CNS myelin extracts, knockdown of AMIGO3 promoted signifcant DRGN neurite outgrowth^8^ and that knockdown of AMIGO3 together with co-incident growth stimulation (here by NT3) promoted signficant DC axon regeneration and improved functional recovery. In addition, spinal cord cavitation was reduced and the lesion site was filled with invading cells, suggesting a further therapeutic use of AMIGO3 and NT-3 in spinal cord injury. It remains to be elucidated why knockdown of AMIGO3 suppressed cavitation that normally results after DC injury in rats^33^. One possibility is that since AMIGO3 belongs to a family of adhesion molecules, knocking down AMIGO3 may have limited the influx of inflammatory cells after DC injury and hence reduced inflammation^34^ limiting secondary damage and cavitation^35,36^.

Although ED1^+^ cells were upregulated in the lesion site of shAMIGO3/nt3-treated animals, we did not identify whether these cells were of the M1 (classical activation) or M2 (alternatively activated macrophages) lineage^37^. However, pro-regenerative M2 macrophage lineages are only present for the first week whilst M1 macrophages persist for more than 4 weeks. Therefore, it is unlikely that ED1^+^ cells were pro-regenerative M2 phenyotypes. However, we are unsure why there are more ED1^+^ cells after treatment with shAMIGO3/nt3 and are currently investigating why this is so. Astrocytes on the other hand play multifaceted roles after spinal cord injury. Normally astrocytes are the major component of the glial scar and prevent axon regeneration by forming a physical and chemical barrier at the lesion borders. However, it appears that shAMIGO3/*nt3* treatment promoted astrocyte invasion into the lesion site, transforming the lesion border from a dense network of tightly woven and thickened processes to a more open meshwork, allowing astrocytes to invade the lesion site. It is possible that astrocyte invasion allowed greater axon regeneration in these animals since they are able to restore the extracellular ionic environment, sequester extracellular glutamate and produce neurotrophic factors^38–41^. Astrocytes secrete axon growth inhibitory CSPG but also adhesive extracellular molecules such as laminin that are supportive to axon growth^42–45^. TGFα infusion into the spinal cord lesion site in mouse also allowed similar astrocyte invasion and new cells as was observed by us with shAMIGO3/*nt3* treatment. For example, the centre of the lesion site contained greater number of cells and increased astrocyte invasion, which correlated with increased numbers of GAP43^+^ axons^46^. These results suggest that astrocytes may provide axon growth supportive roles by migrating into the lesion site.

## Conclusion

Our results demonstrated that non-viral plasmid delivery enhanced transduction in all sizes of DRGN, significantly downregulated AMIGO3 and upregulated NT-3 production from DRGN. This reduced cavity formation and promoted axon regeneration and functional recovery in the damaged spinal cord. These results suggest that AMIGO3 might comprise a future therapy to promote axon regeneration and functional recovery after spinal cord injury. Plasmid transfection is likely to be more translational since viruses need at least 2 weeks to start producing effective titres of the shRNA of choice, whereas plasmids begin to express the relevant payload within hours. Moreover, *in vivo*-jetPEI vector is safe and does not induce off-target immune-mediated cytokines. Therefore, the lack of immunogenic responses after *in vivo*-jetPEI injection is to be expected and is a further advantage over viral vectors.

## Methods

### Preparation of CNS myelin extracts

CNS myelin extracts (CME), containing significant titres of Nogo-A, OMgp, MAG and CSPG were prepared according to our previously published methods^21^.

#### *In vitro* experiments

shAMIGO3/NT-3 plasmid construction: Plasmids (control *gfp*, sh*gfp*, shAMIGO3/*gfp*, nt3/*gfp*, shAMIGO3/*nt3*) were prepared as described previously^47^. To construct bi*cis*tronic plasmids (pH1-shAMIGO3-CMV-*gfp* (shAMIGO3/*gfp*) and pH1-shAMIGO3-CMV-*nt3* (shAMIGO3/*nt3*), shAMIGO3 encoding complementary DNA sequences was designed using the online Ambion software and oligonucleotides were hybridized together and cloned into pRNAT-H1.1/shuttle (Addgene, Cambridge, MA, USA). To create the secretable form of NT3, pCMV-*nt3*-*gfp* (*nt3*/*gfp*), the coding sequence of rat NT3 and the nerve growth factor secretion signal sequence^48^ were cloned into pIRES-EGFP (Clonetech, Mountain View, CA, USA). The efficiency of plasmids to knock down AMIGO3 gene expression was optimised using quantitative real-time PCR (qRT-PCR).

DRGN cultures: Adult DRGN cultures were prepared as described by us earlier^21^. DRGN were cultured in Neurobasal-A (Invitrogen, Paisley, UK) at a plating density of 500/well in chamber slides (Beckton Dickinson, Oxford, UK) pre-coated with 100 μg/ml poly-D-lysine (Sigma, Poole, UK).

*In vivo*-jetPEI (PEI; Polyplus Transfection, New York, USA) was prepared according to the manufacturer’s instructions. DRGN were transfected with 0.5, 1, 2, 3 and 4 μg of plasmid DNA containing either a control sh*gfp* (PEI-sh*gfp*) or shAMIGO3/*nt3* (PEI-shAMIGO3/*nt3*). Additional controls included untreated DRGN (Untreated), DRGN transfected with *in vivo*-jetPEI only (Sham), and shAMIGO3/*gfp* (PEI-shAMIGO3/*gfp*) groups. DRGN were allowed to incubate for 3d before harvesting of cells and extraction of total RNA for validation of shAMIGO3 knockdown using qRT-PCR, as described below.

To monitor NT3 production and disinhibited DRGN neurite outgrowth after shAMIGO3 knockdown and NT3 stimulation, DRGN were transfected with the optimal dose of plasmid DNA (2 μg) and incubated for 3d in the presence of 100 μg/ml CME^21^, before harvesting culture supernatant to detect NT3 production by ELISA and neurite outgrowth by immunocytochemistry.

Quantitative real-time PCR to confirm shAMIGO3 knockdown: Total RNA from treated DRGN (n = 6 wells/treatment) was prepared using Trizol according to the manufacturer’s instructions (Invitrogen). AMIGO3 knockdown and *nt3* over-expression were validated, using qRT–PCR from complementary DNA prepared from extracted mRNA, with appropriate forward and reverse primers (Table 1) using the LightCycler real time qRT-PCR system (Roche, Burgess Hill, UK) according to our previously published methods^19^. Fold-changes were computed using the ΔΔCt method.

**Table 1:** Primers used in this study for qRT-PCR.

Rat genes	Forward (5′-3′)	Reverse (5′-3′)
AMIGO3	CGGCTGCGTGCCTTGTACCT	AGCACTTAGGCCCCGCTGGT
NT3	GCCCCCTCCCTTATACCTAATG	CATAGCGTTTCCTCCGTGGT
TNF-α	ACCACGCTCTTCTGTCTACTG	CTTGGTGGTTTGCTACGAC
IFN-γ	AGGATGCATTCATGAGCATCGCC	CACCGACTCCTTTTCCGCTTCCT
IL-6	TCTCTCCGCAAGAGACTTCCA	ATACTGGTCTGTTGTGGGTGG
IL-12	AATGTTCCAGTGCCTCAACCA	GATCAATCTCTTCGGAAGTGCA
IL-1β	GCAATGGTCGGGACATAGTT	AGACCTGACTTGGCAGAGGA
IFN-β	CGTTCCTGCTGTGCTTCTC	TGTAACTCTTCTCCATCTGTGAC
MX1	AACCCTGCTACCTTTCAA	AAGCATCGTTTTCTCTATTTC
IFIT	CTGAAGGGGAGCGATTGATT	AACGGCACATGACCAAAGAGTAGA
OAS1	TTCTACGCCAATCTCATCAGTG	GGTCCCCCAGCTTCTCCTTAC
Casp7	CAACGACACCGACGCTAATC	GGTCCTTGCCATGCTCATTC
GAPDH	AGACAGCCGCATCTTCTTGT	CTTGCCGTGGGTAGAGTCAT

NT-3 ELISA: NT-3 was assayed in supernatant from n = 6 wells/treatment using the NT-3 DuoSet ELISA kit according to the manufacturer’s instructions (R&D Systems, Abingdon, UK).

Immunocytochemistry: Cells were fixed in 4% paraformaldehyde, washed in 3 changes of PBS before immunocytochemistry as described by us previously^21^. To visualise neurites, DRGN were stained with monoclonal anti-βIII tubulin (Sigma) detected with Alexa-488 anti-mouse antibodies (Invitrogen). Slides were then viewed with an epi-fluorescent Axioplan 2 microscope, equipped with an AxioCam HRc and running Axiovision Software (all from Carl Zeiss, Hertfordshire, UK). The proportion of DRGN with neurites and mean neurite lengths were calculated using Axiovision Software, as previously described^21^. All experiments were performed in duplicate and repeated on 3 independent occasions (i.e. n = 6 wells/condition).

#### *In vivo* experiments

Surgical procedures: All animal surgeries were carried out in strict accordance to the guidelines of the UK Animals Scientific Procedures Act, 1986 and the Revised European Directive 1010/63/EU and conformed to the guidelines and recommendation of the use of animals by the Federation of the European Laboratory Animal Science Associations. Experiments were licensed by the UK Home Office and all experimental protocols were approved by the University of Birmingham’s Animal Welfare and Ethical Review Board. Adult female Sprague-Dawley rats weighing 170–220 g (Charles River, Margate, UK) were used in all experiments. The experiments comprised 8 groups (n = 12 rats/group), including 4 control groups comprising: Intact controls (Group 1: IC; to detect baseline levels), partial laminectomy but no DC lesion; Group 2: sham; to detect surgery-induced changes), DC transected controls (Group 3: DC; to detect injury-mediated changes), and DC + intra-DRG injection of *in vivo-*jetPEI*-gfp* (Group 4: DC + PEI-*gfp;* to monitor DRGN transduction efficiencies) and 4 experimental groups in which DC were transected and DRG injected with plasmid-containing *in vivo*-jetPEI complexes, comprised of: DC + intra-DRG injection of *in vivo-*jetPEI*-gfp* (Group 1: DC + PEI-*gfp);* DC + intra-DRG injection of *in vivo-*jetPEI*-nt3/gfp* (Group 2: DC + PEI*-nt3/gfp*; to monitor DRGN transduction and NT3 over-expression); *DC* + *in vivo-*jetPEI*-*shAMIGO3/*gfp* (Group 3: PE*I-*shAMIGO3*/gfp;* to monitor AMIGO3 knockdown in DRGN and to establish if AMIGO3 knockdown without concomitant stimulation with NT3 promoted DRGN axon regeneration); and DC + intra-DRG injection of *in vivo-*jetPEI*-*shAMIGO3/*nt3* (Group 4: DC + PEI*-*shAMIGO3*/nt3;* to determine if AMIGO3 knockdown and simultaneous stimulation with NT3 was required for DRGN axon regeneration).

Before surgery, rats were injected subcutaneously with 0.05 ml Buprenorphine and anaesthetised using 5% of Isoflurane in 1.8 ml/l of O_2_ with body temperature and heart rate monitored throughout surgery. After partial T8 laminectomy, DC were crushed bilaterally using calibrated watchmaker’s forceps and, after injection of *in vivo* jetPEI/plasmids into L4/L5 DRG^28,33^, rats were killed at either 28d (western blot, immunohistochemistry, qRT-PCR and ELISA) or 6 weeks (electrophysiology and functional tests) in rising concentrations of CO_2_.

Analysis of off-target cytokines and innate immune response genes: Total RNA from n = DRG/treatment were extracted using Trizol reagent as described above. A selection off-target cytokines and innate immune response genes (Table 1) were analysed by qRT-PCR from complementary DNA prepared from extracted mRNA as described above^19^. Fold-changes were computed using the ΔΔCt method.

Tissue preparation and cryo-sectioning: Rats were intracardially perfused with 4% formaldehyde and DRG and segments of T8 cord containing the DC injury sites were post-fixed for 2 h at RT, prepared for cryoprotection and sectioned using a cryostat as described by us previously^33^.

Immunohistochemistry: Immunohistochemistry was performed on sections from the middle of DC lesion sites or middle of the DRG as described previously^8,21,33^. Primary antibodies included: anti-AMIGO3 (1:200 dilution; Santa Cruz Biotechnology, San Diego, CA, USA), anti-GAP43 (1:400 dilution; Zymed, San Francisco, CA, USA); anti-NT3 (1:400 dilution; Abcam, Cambridge, UK); anti-GFAP (1:400 dilution, Sigma); anti-CD68 (1:500 dilution, Abcam) and anti-rat ED1 (1:400 dilution; Serotec, Oxford, UK).

Protein extraction, western blot and densitometry: Total protein from DRG was extracted and western blot followed by densitometry was performed according to our previously published methods^49,50^. Briefly, 40 µg of total protein extract was resolved on 12% SDS gels, transferred to polyvinylidene fluoride (PVDF) membranes (Millipore, Watford, UK) and probed with relevant primary antibodies: anti-AMIGO3 (1:400 dilution, Santa Cruz); anti-NT-3 (1:1000 dilution, Abcam) and β-actin (1:1000 dilution, Sigma). Membranes were then incubated with relevant HRP-labelled secondary antibodies and bands were detected using the enhanced chemiluminescence kit (GE Healthcare, Buckinghamshire, UK). Anti β-actin antibodies were used as a protein loading control.

For densitometry, western blots were scanned into Adobe Photoshop (Adobe Systems Inc, San Jose, CA, USA) keeping all scanning parameters the same between blots and the integrated density of bands analysed using the built-in-macros for gel analysis in ImageJ (NIH, USA, http://imagej.nih.gov/ij)^21,50–52^. Means ± SEM were plotted in Microsoft Excel (Microsoft Corporation, CA, USA).

Analysis of GFP expression: Images of sections (n = 160) of L4/L5 DRG from each animal (n = 10) were captured at x10 magnification using the Zeiss Axioplan 200 (Carl Zeiss). Images were merged in Adobe Photoshop (Adobe Systems Incorporated) using Photomerge and the total number of GFP^+^ DRGN and their diameters recorded, as described previously^28^.

Quantification of axons: Axon regeneration in the spinal cord was quantified according to previously published methods^53^. Briefly, serial parasagittal sections of cords were reconstructed by collecting all serial 50 μm-thick sections (∼70–80 sections/animal; n = 10 rats/treatment). In each section, the numbers of intersections of GAP43^+^ fibers through a dorsoventral orientated line was counted from 4 mm rostral to 4 mm caudal to the lesion site. Axon number was calculated as a percentage of the fibers seen 4 mm above the lesion, where the DC was intact.

Electrophysiology: Four weeks after surgery or treatment, compound action potentials (CAP) were recorded after shAMIGO3/nt3 treatment as previously described^54,55^. The experimenter was blinded to the treatment status of the animals. The amplitude of CAP was calculated between the negative deflection after the stimulus artifact and the next peak of the wave. The area of the CAP was calculated by rectifying the CAP component (full-wave rectification) and measuring its area. At the end of each experiment, the dorsal half of the spinal cord was transected between stimulating and recording electrodes to confirm that a CAP could not be detected.

#### Functional tests

Functional testing after DC lesions was carried out as described by others^20^. Animals (n = 18/group) were trained to master traversing the rope and ladder for 1w before functional testing. All functional tests were performed 2-3d before injury to establish baseline parameters. Animals were then tested 2d after DC lesion + treatment and then weekly for 6 weeks. Experiments were performed by 2 observers (from which the treatment conditions were masked) in the same order, the same time of day and each test performed for 3 individual trials.

Horizontal ladder test: This tests the animals locomoter function and is performed on a 0.9-meter-long horizontal ladder with a diameter of 15.5 cm and randomly adjusted rungs with variable gaps of 3.5–5.0 cm. Animals were assessed traversing the ladder and the left and right rear paw slips were recorded along with the total number of steps. To calculate the mean error rate: the number of slips were divided by the total number of steps.

Tape removal test (sensory function): The tape removal test determines touch perception from the left hind paw. Animals were held with both hind-paws extended and a piece of tape of 15 × 15 mm (Kip Hochkrepp, Bocholt, Germany) was affixed to the palm of the left hind-paw. The time it took for the animals to detect and remove the tape was recorded and used to calculate the mean sensing time.

### Statistical analysis

Data are presented as means ± SEM. Significant differences were calculated using SPSS Version 22 (IBM, NJ, USA) software by one-way analysis of variance (ANOVA), with Bonferroni *post hoc* tests, set at P < 0.05.

For the horizontal ladder crossing functional tests, data was analysed according to Fagoe *et al*.^20^ using R package (www.r-project.org). Whole time-course of lesioned and sham-treated animals were compared using binomial generalized linear mixed models (GLMM), with: lesion/sham set to true in lesioned animals post-surgery (set to false otherwise); operated/unoperated set to false before surgery, true after surgery as fixed factors; animals as random factors and time as a continuous covariate. Binomial GLMMs were fitted in R using package *lme4* with the *glmer* function. P values were then calculated using parametric bootstrap. For the tape removal test, linear mixed models (LMM) were calculated by model comparison in R using the package *pbkrtest*, with the Kenward-Roger method^20^.

### Data availability

All data generated or analysed during this study are included in this article.
